# Supplementary material for: Impact of Lactobacillus- and Bifidobacterium-Based Direct-Fed Microbials on the Performance, Intestinal Morphology, and Fecal Bacterial Populations of Nursery Pigs
Source: Microorganisms. 2024 Aug 28;12(9):1786. doi: 10.3390/microorganisms12091786 (PMC11433873; doi:10.3390/microorganisms12091786)
Supplement: Supplementary file 1 [file microorganisms-12-01786-s001.zip › Supplementary Material Document S1.pdf]

Diet Composition (kg) Phases 1-2

	Phase 1				Phase 2			
	Control	0.1% LacPro	0.2% LacPro	0.2% BifPos	Control	0.1% LacPro	0.2% LacPro	0.2% LacPro
Corn	212	211.2	211.2	211.2	433.8	432.9	432.9	432.9
Soybean Meal					137.4	137.4	137.4	137.4
DDGS					45.4	45.4	45.4	45.4
Monocalcium Phosphate 21%	5.8	5.8	5.8	5.8	8.7	8.7	8.7	8.7
Limestone	7.9	7.9	7.9	7.9	7.9	7.9	7.9	7.9
Corn Oil	5.9	5.9	5.9	5.9	5.7	5.7	5.7	5.7
Lysine HCL	5.4	5.4	5.4	5.4	5.4	5.4	5.4	5.4
Salt	2.3	2.3	2.3	2.3	4.1	4.1	4.1	4.1
Zinc Oxide	3.6	3.6	3.6	3.6	2.7	2.7	2.7	2.7
Threonine PRO 80%	2.9	2.9	2.9	2.9	2.7	2.7	2.7	2.7
DL-Methionine-99%	2.7	2.7	2.7	2.7	2.3	2.3	2.3	2.3
L-Valine	0.2	0.2	0.2	0.2	0.6	0.6	0.6	0.6
L-Tryptophan	0.5	0.5	0.5	0.5	0.5	0.5	0.5	0.5
PGF VTM[1]					0.5	0.5	0.5	0.5
Premix*		0.9	0.9	0.9		0.9	0.9	0.9
1200 Crumble[2]	544	544	544	544	249.5	249.5	249.5	249.5
Steam Rolled Oats	113	113	113	113				

[1]Provided per kilogram of the diet: 1,998 FTU phytase, 3,522 IU vitamin A, 1,101 IU vitamin D3, 22 IU vitamin E, 3.0 mg vitamin K3, 26.4 mg niacin,
17.6 mg pantothenic acid, 5.2 mg riboflavin, 23.8 ug vitamin B12, 30 mg Mn from manganous oxide, 100 mg Zn from zinc hydroxychloride, 80 mg Fe
from ferrous sulfate, 12 mg Cu from copper chloride, 0.40 mg I from ethylenediamine dihydroiodide, and 0.30 mg Se from sodium selenite.

[2]Whey permeate, soybean meal, Soy Protein, Porcine Specialty Protein, steamed rolled oats, Fat, Biological Protein, L-Valine, VTM

*Premix contained ground corn and the *Lactobacillus*-based probiotic added to provide the final diets with 0.1% or 0.2% or final diets with 0.2% *Bifidobacteria*-based postbiotic. Premix was mixed in the SDSU animal science department. Premix was prepared and delivered with every dietary phase change, batches of ground corn were weighed as (0.9 kg) of the total amount for the phase.

Diet Composition (kg) Phases 3-4

	Phase 3				Phase 4			
	Control	0.1% LacPro	0.2% LacPro	0.2% BifPos	Control	0.1% LacPro	0.2% LacPro	0.2% BifPos
Corn	518.6	517.7	517.7	517.7	574.2	574.2	574.2	574.2
Soybean Meal	247.2	247.2	247.2	247.2	207.7	207.7	207.7	207.7
DDGS	90.7	90.7	90.7	90.7	90.7	90.7	90.7	90.7
Corn Oil	17.6	17.6	17.6	17.6	5.4	5.4	5.4	5.4
Limestone	8.4	8.4	8.4	8.4	9.5	9.5	9.5	9.5
Monocalcium Phosphate 21%	7.7	7.7	7.7	7.7	3.6	3.6	3.6	3.6
Salt	5.4	5.4	5.4	5.4	4.5	4.5	4.5	4.5
Lysine HCL	5.2	5.2	5.2	5.2	4.9	4.9	4.9	4.9
Threonine PRO 80%	2.4	2.4	2.4	2.4	2.2	2.2	2.2	2.2
DL-Methionine-99%	1.8	1.8	1.8	1.8	1.5	1.5	1.5	1.5
PGF VTM[1]	0.9	0.9	0.9	0.9	0.9	0.9	0.9	0.9
L-Valine	0.5	0.5	0.5	0.5	0.4	0.4	0.4	0.4
L-Tryptophan	0.5	0.5	0.5	0.5	0.5	0.5	0.5	0.5
Premix*		0.9	0.9	0.9				

[1]Provided per kilogram of the diet: 1,998 FTU phytase, 3,522 IU vitamin A, 1,101 IU vitamin D3, 22 IU vitamin E, 3.0 mg vitamin K3, 26.4 mg niacin,
17.6 mg pantothenic acid, 5.2 mg riboflavin, 23.8 ug vitamin B12, 30 mg Mn from manganous oxide, 100 mg Zn from zinc hydroxychloride, 80 mg Fe
from ferrous sulfate, 12 mg Cu from copper chloride, 0.40 mg I from ethylenediamine dihydroiodide, and 0.30 mg Se from sodium selenite.

Micronutrients: (Copper Chloride at 0.3 kg for Phase 4 and 0.2 kg for Phase 5)

*Premix contained ground corn and the *Lactobacillus*-based probiotic added to provide the final diets with 0.1% or 0.2% or final diets with 0.2% *Bifidobacteria*-based postbiotic. Premix was mixed in the SDSU animal science department. Premix was prepared and delivered with every dietary phase change; batches of ground corn were weighed (0.9 kg) of the total amount of the diet for the phase.

Diet Composition (kg) Phase 5-6

	Phase 5				Phase 6			
	Control	0.1% LacPro	0.2% LacPro	0.2% BifPos	Control	0.1% LacPro	0.2% LacPro	0.2% LacPro
Corn	614.7	614.7	614.7	614.7	661.1	661.1	661.1	661.1
Soybean Meal	168.7	168.7	168.7	168.7	124.3	124.3	124.3	124.3
DDGS	90.7	90.7	90.7	90.7	90.7	90.7	90.7	90.7
Corn Oil	5.2	5.2	5.2	5.2	9.3	9.3	9.3	9.3
Limestone	9.5	9.5	9.5	9.5	5	5	5	5
Monocalcium Phosphate 21%	3.4	3.4	3.4	3.4	4.5	4.5	4.5	4.5
Salt	4.5	4.5	4.5	4.5	4.3	4.3	4.3	4.3
Lysine HCL	4.6	4.6	4.6	4.6	3	3	3	3
Threonine PRO 80%	1.9	1.9	1.9	1.9	1.7	1.7	1.7	1.7
DL-Methionine-99%	1.1	1.1	1.1	1.1	0.9	0.9	0.9	0.9
PGF VTM[1]	0.9	0.9	0.9	0.9	0.7	0.7	0.7	0.7
L-Valine	0.2	0.2	0.2	0.2	0.4	0.4	0.4	0.4
L-Tryptophan	0.4	0.4	0.4	0.4	0.2	0.2	0.2	0.2

[1]Provided per kilogram of the diet: 1,998 FTU phytase, 3,522 IU vitamin A, 1,101 IU vitamin D3, 22 IU vitamin E, 3.0 mg vitamin K3, 26.4 mg niacin,
17.6 mg pantothenic acid, 5.2 mg riboflavin, 23.8 ug vitamin B12, 30 mg Mn from manganous oxide, 100 mg Zn from zinc hydroxychloride, 80 mg Fe
from ferrous sulfate, 12 mg Cu from copper chloride, 0.40 mg I from ethylenediamine dihydroiodide, and 0.30 mg Se from sodium selenite.
Micronutrients: (Copper Chloride at 0.2 kg for Phase 6 and 0.04 kg for Phase 7)

Diet Composition (kg) Phase 7-8

	Phase 7				Phase 8			
	Control	0.1% LacPro	0.2% LacPro	0.2% BifPos	Control	0.1% LacPro	0.2% LacPro	0.2% LacPro
Corn	714.4	714.4	714.4	714.4	738.4	738.4	738.4	738.4
Soybean Meal	108.4	108.4	108.4	108.4	97.1	97.1	97.1	97.1
DDGS	56.7	56.7	56.7	56.7	45.4	45.4	45.4	45.4
Corn Oil	8.6	8.6	8.6	8.6	8.2	8.2	8.2	8.2
Limestone	3.8	3.8	3.8	3.8	4.1	4.1	4.1	4.1
Monocalcium Phosphate 21%	4.5	4.5	4.5	4.5	4.5	4.5	4.5	4.5
Salt	3.9	3.9	3.9	3.9	3.6	3.6	3.6	3.6
Lysine HCL	3.2	3.2	3.2	3.2	2.6	2.6	2.6	2.6
Threonine PRO 80%	1.5	1.5	1.5	1.5	1.4	1.4	1.4	1.4
DL-Methionine-99%	0.5	0.5	0.5	0.5	0.4	0.4	0.4	0.4
PGF VTM	0.9	0.9	0.9	0.9	0.9	0.9	0.9	0.9
L-Valine								
L-Tryptophan	0.4	0.4	0.4	0.4	0.3	0.3	0.3	0.3

[1]Provided per kilogram of the diet: 1,998 FTU phytase, 3,522 IU vitamin A, 1,101 IU vitamin D3, 22 IU vitamin E, 3.0 mg vitamin K3, 26.4 mg niacin,
17.6 mg pantothenic acid, 5.2 mg riboflavin, 23.8 ug vitamin B12, 30 mg Mn from manganous oxide, 100 mg Zn from zinc hydroxychloride, 80 mg Fe
from ferrous sulfate, 12 mg Cu from copper chloride, 0.40 mg I from ethylenediamine dihydroiodide, and 0.30 mg Se from sodium selenite.

Micronutrients (Copper Chloride): 0.2 kg

Diet Composition (kg) Phase 9

	Phase 9			
	Control	0.1% LacPro	0.2% LacPro	0.2% BifPos
Corn	792	792	792	792
Soybean Meal	89.4	89.4	89.4	89.4
DDGS				
Corn Oil	4.1	4.1	4.1	4.1
Limestone	7.7	7.7	7.7	7.7
Monocalcium Phosphate 21%	3.6	3.6	3.6	3.6
Salt	4.5	4.5	4.5	4.5
Lysine HCL	2.9	2.9	2.9	2.9
Threonine PRO 80%	1.1	1.1	1.1	1.1
DL-Methionine-99%	0.1	0.1	0.1	0.1
PGF VTM	0.9	0.9	0.9	0.9
L-Valine				
L-Tryptophan	0.3	0.3	0.3	0.3

[1]Provided per kilogram of the diet: 1,998 FTU phytase, 3,522 IU vitamin A, 1,101 IU vitamin D3, 22 IU vitamin E, 3.0 mg vitamin K3, 26.4 mg niacin,
17.6 mg pantothenic acid, 5.2 mg riboflavin, 23.8 ug vitamin B12, 30 mg Mn from manganous oxide, 100 mg Zn from zinc hydroxychloride, 80 mg Fe
from ferrous sulfate, 12 mg Cu from copper chloride, 0.40 mg I from ethylenediamine dihydroiodide, and 0.30 mg Se from sodium selenite.
Micronutrients: (Copper Chloride at 0.2 kg)
